# Polypharmacy and frailty among aging World Trade Center responders

**DOI:** 10.1371/journal.pone.0337391

**Published:** 2025-12-04

**Authors:** Chinmayi Venkatram, Fred Ko, Ghalib Bello, Ahmad Sabra, Hannah M. Thompson, Erin Thanik, Elena Colicino, Roberto G. Lucchini, Michael Crane, Susan L. Teitelbaum, Katherine A. Ornstein, William W. Hung

**Affiliations:** 1 Department of Medical Education, Icahn School of Medicine at Mount Sinai, New York, New York, United States of America; 2 Brookdale Department of Geriatrics and Palliative Medicine, Icahn School of Medicine at Mount Sinai, New York, New York, United States of America; 3 Geriatric, Research, Education and Clinical Center, James J Peters VA Medical Center, Bronx, New York, United States of America; 4 Department of Environmental Medicine and Public Health, Icahn School of Medicine at Mount Sinai, New York, New York, United States of America; 5 General Responder Data Center, Environmental Medicine and Public Health, Icahn School of Medicine at Mount Sinai, New York, New York, United States of America; 6 Department of Environmental Health Sciences, School of Public Health, Florida International University, Miami, Florida, United States of America; 7 Johns Hopkins School of Nursing, Baltimore, Maryland, United States of America; Yarmouk University, JORDAN

## Abstract

**Background:**

During and after the 9/11 rescue and recovery efforts, World Trade Center (WTC) responders were exposed to environmental hazards that may accelerate aging and increase frailty. This study examines the relationship between polypharmacy and frailty among WTC responders to inform strategies that mitigate medication-related risks in high-risk, aging populations.

**Methods:**

We included WTC responders aged 50 and older who attended at least one clinical monitoring visit at WTC Health Program between 2017–2019. Frailty was assessed using the WTC-specific Clinical Frailty Index, and associations with polypharmacy (concurrent use of 5 or more medications) and fall-risk increasing drugs (FRIDs) use were evaluated through multivariable logistic regression models adjusting for demographic, employment, health, and WTC exposure data.

**Results:**

Among 6,966 WTC responders, 55% met the criteria for polypharmacy and 7.6% used FRIDs. Frailty was independently associated with both polypharmacy (OR 1.15, p < 0.001) and FRID use (OR 1.11, p < 0.001). Older age (OR 1.08, p < 0.001), obesity (OR 1.92, p < 0.001 for BMI ≥ 30), protective service occupations (OR 1.30, p = 0.002), and chronic conditions such as gastroesophageal reflux disease (OR 1.71, p < 0.001), obstructive airway disease (OR 2.24, p < 0.001), and upper respiratory disease (OR 1.85, p < 0.001) were associated with higher odds of polypharmacy. In contrast, male sex (OR 0.81, p = 0.018) and construction occupations (OR 0.73, p = 0.001) were associated with lower odds of polypharmacy. Female sex (OR 1.64, p < 0.001), smoking (current: OR 1.55, p = 0.013; former: OR 1.30, p = 0.014), and mental health conditions such as anxiety (OR 1.66, p = 0.004), depression (OR 2.85, p < 0.001), and post-traumatic stress disorder (OR 1.72, p < 0.001) were associated with higher odds of FRID use.

**Conclusions:**

We found a high prevalence of polypharmacy and FRID use among aging WTC responders, with frailty significantly associated with both. Our findings underscore the need to optimize medication management for aging WTC responders, which may impact their healthy aging.

## Introduction

By 2030, the majority of World Trade Center (WTC) rescue and recovery workers (termed responders) estimated at over 91,000 individuals, will be aged 65 and over, placing them at increased risk for age-related conditions [1,2]. These responders were exposed to high levels of toxicants and experienced intense psychological trauma during the emergency response and cleanup following the 2001 WTC disaster [3]. Such exposures have been known to contribute to various health conditions and may accelerate the aging process, heightening the risk of chronic diseases and age-related syndromes [4,5]. Currently, the most common conditions among these responders, as recorded by the World Trade Center Health Programs (WTCHPs), are aerodigestive illnesses (45%), cancer (16%), and mental health issues (16%) [6]. Among age-related syndromes, frailty is of particular concern given the overall chronological age of the WTC responders as well as the high burden of disease, both of which could contribute to negative health outcomes [7,8]. Frailty is a geriatric syndrome that is characterized by decreased physiological reserve and increased vulnerability to stressors which has been associated with poor outcomes including morbidity and death [9].

Polypharmacy, commonly defined as the use of five or more medications, is prevalent among older adults across the U.S., with approximately 36.8–44.1% of individuals aged 65 and older reporting this level of medication use [10–14]. Polypharmacy has been linked to an increased risk of adverse health outcomes, particularly in older adults, as age-related changes in drug metabolism and physiology make them more susceptible to adverse drug reactions [15]. Given their increased risk of chronic conditions, WTC responders are likely to require treatment with multiple medications, which can increase their vulnerability to polypharmacy and its associated risks, including the need for medications such as anticholinergics and psychotropics. Known as Falls Risk Increasing Drugs (FRIDs), the benefits of these medications may not outweigh the risks for older adults due to their adverse effects, limited therapeutic benefits, and the availability of safer alternatives [16–18].

These issues are particularly relevant for WTC responders, as recent findings suggest that frailty is associated with higher levels of WTC exposure severity— a composite measure of the responders’ experiences and exposure— contributing to the earlier onset of chronic diseases [7]. Although frailty and polypharmacy are well-documented concerns in aging populations, evidence suggests a strong association between the two, with polypharmacy serving as both a contributor to frailty and a consequence of its progression due to increased medication needs [19]. Despite this, no studies have specifically explored these factors in WTC responders, a group exposed to unique environmental hazards. As this cohort continues to age, identifying factors contributing to early aging and potential intervention points is crucial. The goal of this study was to examine patterns of polypharmacy and FRID use in the aging WTC responder cohort. We also evaluated frailty and other cohort characteristics to identify factors associated with these outcomes. Our findings could inform strategies to manage polypharmacy and prevent frailty in high-risk, aging populations exposed to toxicants.

## Materials and methods

### Study design and population

Following the 9/11 World Trade Center disaster, the National Institute for Occupational Safety and Health (NIOSH) established the WTCHP to provide responders with yearly health examinations and tests (termed monitoring) as well as treatment for specific WTC-related conditions, which are health conditions certified by the WTCHP deemed related to responders’ efforts at the WTC [20,21]. This study focuses on a subset of non-firefighter responders in the WTCHP (General Responder Cohort (GRC) beginning in 2002. Details on this cohort are described elsewhere [20]. The WTCHP obtained informed consent from all participants via a standardized form approved by the Mount Sinai Institutional Review Board (IRB).

**Fig 1:**
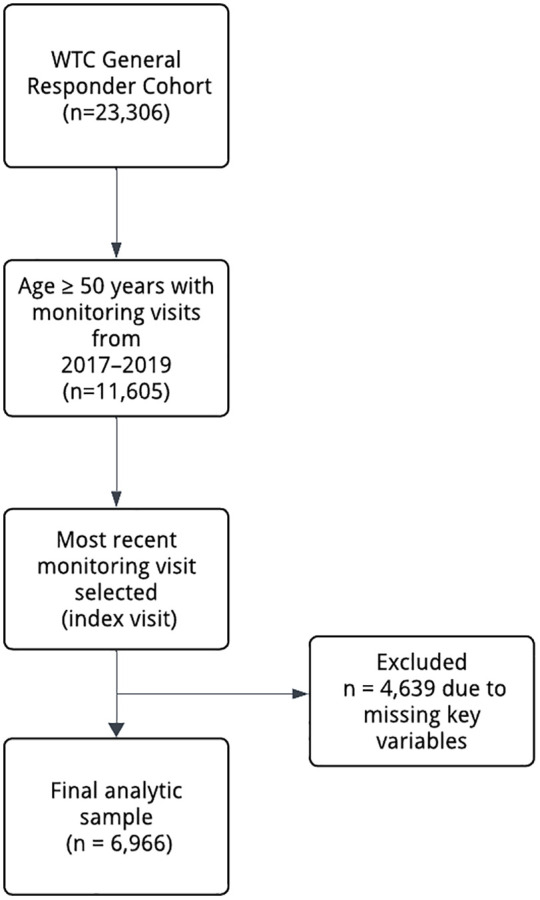
Flowchart of participant inclusion and exclusion for the analytic sample.

This cross-sectional study analyzed patterns of polypharmacy and FRID use, and examined their associations with frailty and related health factors among members of the WTC-GRC who underwent annual monitoring at the Mount Sinai Selikoff Centers for Occupational Health in New York City, the largest WTCHP Clinical Center of Excellence. We restricted our study to responders whose monitoring visits were conducted at this location to ensure consistency in data collection (n = 23,306). We included responders aged 50 years and older who attended at least one monitoring visit between 2017 and 2019, choosing this period to capture the most recent pre-COVID pandemic data (n = 11,605). Among those with multiple visits during the study time period, we selected only the most recent visit for each participant, referred to here as the “index visit,” to provide a consistent point of reference. Responders with missing data on key variables—including self-reported medication use, smoking, alcohol use, body mass index (BMI), exposure severity, demographics, and WTC Clinical Frailty Index (WTC FI-Clinical) items —were excluded from the analysis (n = 4,639), resulting in a final study sample of 6,966 participants. Fig 1 presents the flow of participants in the study, from the initial WTC-GRC to the final analytic sample after applying selection criteria.

### Data collection and sources

Data collected during the annual monitoring visits as part of the WTCHP were utilized for this study. These visits incorporate several standardized assessments [20]. At their first visit, participants completed the Exposure Assessment Questionnaire (EAQ), which documents the extent of their exposure to pollutants at the WTC site. The Interviewer-Administered Medical Questionnaire (IAMQ) and the Self-Administered Mental Health Questionnaire (SAMHQ) are also administered at the first visit and at all subsequent visits, gathering data on medical history, medication use, and health behaviors, mental health status and functional limitations.

Data were accessed for research purposes on September 14th, 2021. Throughout the study, authors did not have access to information that could identify individual participants during or after data collection.

### Key measures

#### Demographic and pre-9/11 occupation.

Demographic information included age, sex, and race/ethnicity. We categorized participants’ pre-9/11 occupations as: construction; protective services (e.g., police and emergency medical services); cleaning, maintenance, installation, and repair; and other occupations, which encompassed roles in management, legal, sales, and additional fields not listed above.

#### WTC exposure severity.

WTC exposure severity were categorized as low, intermediate, and high or very high [22]. These categories were determined based on duration of work on the WTC cleanup effort, whether the individual was exposed to the dust cloud on 9/11, and whether they worked on the debris pile.

#### Medication use (polypharmacy and FRIDs).

We documented self-reported medication use through IAMQ responses, including prescription and non-prescription drugs. Responders were asked, “Are you currently taking any prescription, non-prescription or herbal medications?” If responders answered ‘Yes,’ they were prompted to list all medications, including alternative, herbal, homeopathic, or other supplements. For this study, the total medication count was determined by the number of medications listed; if none were listed, the count was zero. We defined polypharmacy as the concurrent use of five or more medications and hyper-polypharmacy as ten or more [23].

We identified FRIDs based on specific drug classes linked to fall risk: benzodiazepines, first-generation antihistamines, long-acting hypoglycemics, skeletal muscle relaxants, and tertiary tricyclic antidepressants [24,25]. To classify these medications, we used a string-matching algorithm (COMPGED function in SAS) that computes the generalized edit distance between two strings, allowing for matches between recorded medications and the list of brand and generic names in each FRID class.

#### Health characteristics.

We included WTC-certified conditions in our analysis. Fourteen certification categories were available in the dataset; however, we excluded categories with low frequencies (fewer than 250 cases). The analysis focused on the following WTC-certified conditions: anxiety disorder, cancer, depression, gastroesophageal reflux disease (GERD), obstructive airway disease (OAD), post-traumatic stress disorder (PTSD), and upper respiratory disease (URD). Additionally, we included three self-reported medical conditions from the IAMQ: sleep apnea, diabetes, and hypertension. These were selected from the IAMQ’s list of longitudinal conditions based on their frequency and relevance to the study. Other health-related variables incorporated in the analysis included BMI, smoking status, and alcohol use. BMI was categorized as <25, 25–30, and >30, corresponding to underweight/normal, overweight, and obesity, respectively. Smoking status was self-reported via the IAMQ and categorized as never, former, or current smoker. Participants were asked (1) whether they had smoked at least 100 cigarettes (five packs) in their lifetime and (2) whether they currently smoke cigarettes as of the past week. Those who reported never smoking 100 cigarettes were categorized as never smokers. Those who reported smoking ≥100 cigarettes in their lifetime but not currently smoking were categorized as former smokers. Those who reported currently smoking were categorized as current smokers. Alcohol use was categorized as none/nondrinker, less than one drink per week, and one or more drinks per week.

#### Self-reported health and functional limitations.

Self-reported health was assessed from the SAMHQ question asking participants to rate their overall health as excellent, very good, good, fair, and poor. For analysis, we dichotomized the responses as excellent/very good/good and fair/poor. We also chose four self-reported functional limitations from SAMHQ based on their relevance to frailty and overall health outcomes. These limitations included (1) physical health limitations (accomplished less due to physical health; yes/no), (2) short-term memory problems (not at all, a little, moderately, or quite a bit/extremely), (3) pain affecting work (not at all, a little, moderately, or quite a bit/extremely), and (4) the impact of health on moderate activities (yes/no). We analyzed these individual functional limitations as individual variables to further explore their associations with polypharmacy and frailty in the WTC responder cohort.

#### Frailty measurement.

We measured frailty using the WTC FI-Clinical which was developed using data from WTCHP cohort members [7]. This frailty index was constructed using clinical parameters selected from a broad set of potential age-related indicators following a well-established deficit accumulation model of frailty [26]. Thirty parameters were used to construct the original WTC FI-Clinical, with each subject’s frailty score calculated as the proportion of these parameters in which deficits were present. Since medication use was included as one of the original thirty parameters, we recalculated the WTC FI-Clinical after excluding the medication use variable. Scores on the WTC FI-Clinical range from 0 (no deficits present) to 1 (all deficits present), though they typically do not exceed 0.7, which is considered the biological limit of deficit accumulation [27]. For analysis (described below), we modeled frailty using the total deficit count as a continuous predictor.

### Statistical analyses

#### Patterns of polypharmacy and FRID use across cohort characteristics.

We first explored the relationship between polypharmacy and cohort characteristics employing bivariate analyses. For continuous variables, such as cohort age and WTC FI-Clinical, the relationship with polypharmacy was assessed using the Wilcoxon rank sum test. For categorical variables, Pearson’s Chi-squared test was used to evaluate their association with polypharmacy.

We then examined the association of polypharmacy with cohort characteristics using multivariable logistic regression models. In these models, polypharmacy was the dependent variable, and cohort characteristics including frailty (WTC FI-Clinical, modeled continuously) were the independent variables. Independent variables were selected a priori based on prior literature and clinical relevance and were retained in the models regardless of statistical significance given their conceptual and clinical importance in our cohort [7,8,10,19,22]. We fitted a logistic regression model that included the WTC FI-Clinical variable along with other variables not used in constructing the frailty index. WTC FI-Clinical was entered into the models as deficit counts rather than ratios in order to improve interpretability (i.e., odd ratios are per additional deficit). We report all associations in terms of Odds Ratios (OR) and we identified statistical significant results with a 5% type I error.

The same modelling approach outlined above was used to assess the relationship of FRID use (defined as use of one or more FRIDs versus none) with the same set of risk factors listed above.

Because listwise exclusion reduced the analytic sample from 11,605 eligible responders to 6,966 with complete data (see Study Design and Population), we conducted sensitivity analyses to evaluate potential bias from missingness, using multiple imputation by chained equations (MICE) under a Missing at Random (MAR) assumption [28]. Imputation models were specified according to variable type: predictive mean matching for continuous/count variables, logistic for binary, polytomous logistic for nominal factors, and proportional odds for ordered factors [29]. We then refit our multivariable logistic regression models for polypharmacy and FRID use on each imputed dataset (n = 11,605) and pooled results using Rubin’s rules [30].

All data preparation and statistical analyses were performed in R [31].

## Results

Our analytic cohort consisted of 6,966 WTC responders. Baseline characteristics are summarized in Table 1. The median age was 58 years old (interquartile range 54, 63); 86% were male with 62% identified as White, 14% were Black and 21% Hispanic. Regarding the severity of WTC exposure, 14% had low exposure, 63% had intermediate exposure and 23% had high or very high exposure. In the study sample, occupation prior to 9/11/2001 varied, including 20% in construction, 53% in protective service occupations (e.g., police, emergency medical services), 9.4% in cleaning, maintenance, installation or repair, and 18% other (encapsulates occupations not covered by the categories above). Twenty percent of participants had a WTC-certified history of cancer, 38% had GERD, 27% had OAD, and 46% had URD. In addition, non-WTC-certified chronic diseases were prevalent in the cohort, with 21% self-reporting diabetes, 56% reporting hypertension, and 39% reporting sleep apnea. Among WTC-certified mental health conditions, 4.8% of participants had depression, 8.4% had PTSD, and 3.9% had an anxiety disorder. Obesity, defined as BMI at 30 or over, was prevalent in 49% of the cohort. Regarding self-reported health, 33% of participants rated their health as fair or poor, 43% reported that their physical health impacted their work or daily activities, and 22% indicated that pain interfered with their normal work (including outside employment and housework) to a significant or extreme degree. Additionally, 18% reported having significant or extreme difficulty with short-term memory. The median deficit count for frailty (WTC FI-Clinical) was 4.0 with an interquartile range of 1.0 and 8.0, corresponding to a frailty score (deficit count as a proportion of all possible deficits) of 0.14 (0.03–0.28) [7].

**Table 1:** WTC general responder participant characteristics.

Characteristics	N = 6,966
Age at most recent visit, Median (IQR)	58 (54, 63)
Sex	
Female	945 (14%)
Male	6,021 (86%)
Race	
hite	4,351 (62%)
Black	941 (14%)
Hispanic	1,480 (21%)
Other	194 (2.8%)
Enrollment Year	
2002-2005	2,684 (39%)
2006-2008	1,658 (24%)
2009-present	2,624 (38%)
Exposure Severity	
Low	991 (14%)
High/Very High	1,591 (23%)
Intermediate	4,384 (63%)
Pre-9/11 Occupation	
Other	1,268 (18%)
Construction	1,379 (20%)
Protective	3,664 (53%)
Maintenance and Repair	655 (9.4%)
Smoking status	
Never smoker	4,292 (62%)
Current smoker	423 (6.1%)
Former smoker	2,251 (32%)
Alcohol use	
None/non-drinker	1,922 (28%)
Less than one drink per week	3,884 (56%)
More than one drink per week	1,160 (17%)
BMI	
<25	844 (12%)
25-30	2,731 (39%)
>30	3,391 (49%)
Chronic Conditions	
Sleep Apnea	2,722 (39%)
Diabetes	1,495 (21%)
Hypertension	3,928 (56%)
Anxiety Disorder	273 (3.9%)
Cancer	1,363 (20%)
Depression	332 (4.8%)
GERD	2,662 (38%)
Obstructive Airway Disease	1,909 (27%)
PTSD	583 (8.4%)
Upper Respiratory Disease	3,232 (46%)
Self-reported health	
Excellent/very good/good	4,693 (67%)
Fair/poor	2,273 (33%)
*Health limits accomplishments	2,989 (43%)
**Health limits moderate activities	1,087 (16%)
Pain limits work	
Not at all	2110 (30.3%)
A little bit	1779 (25.5%)
Moderately	1536 (22%)
Quite a bit/Extremely	1541 (22.1%)
Short term memory problems	
Not at all	1644 (23.6%)
A little	2688 (38.6%)
Moderately	1352 (19.4%)
Quite a bit/extremely	1282 (18.4%)
WTC FI-Clinical (deficit count)	
Median (IQR)	4.0 (1.0, 8.0)

*“During the past 4 weeks, have you had any of the following problems with your work or regular daily activities as a result of your physical health: Accomplished less than you would like?”.

**“Does your health now limit you in: Moderate activities, such as moving a table, pushing a vacuum cleaner, bowling, or playing golf”.

**Fig 2:**
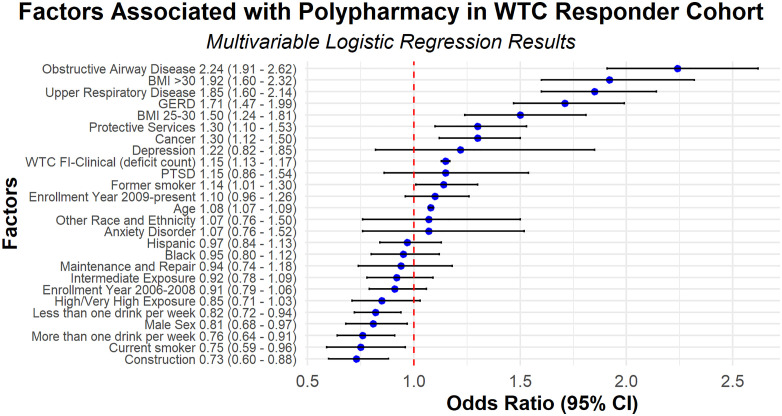
This forest plot displays the results of a multivariable logistic regression analysis of factors associated with polypharmacy in the WTC Responder Cohort. Odds ratios and corresponding 95% confidence intervals are shown for each factor.

At each participant’s index monitoring visit, the mean number of medications used was 6 (SD 5.02), with 55% of the cohort meeting criteria for polypharmacy and 21% for hyper-polypharmacy. Table 2 displays bivariate associations between cohort characteristics and polypharmacy. In the bivariate analysis, frailty was associated with polypharmacy. Polypharmacy was also associated with older age, female sex, occupations in construction, maintenance and repair, and other roles, obesity, smoking, and chronic conditions including hypertension, diabetes mellitus, OAD, sleep apnea, URD, cancer, GERD, PTSD, depression, and anxiety. Fig 2 presents the results of the multivariable model that evaluates factors associated with polypharmacy. In the multivariable model, frailty was independently associated with polypharmacy. Each additional deficit in the WTC FI-Clinical was associated with a 15% increase in the odds of polypharmacy (Odds Ratio (OR) 1.15; 95% Confidence Interval (CI) 1.13, 1.17). There were significant associations between several cohort characteristics and polypharmacy. Each additional year of age was associated with an 8% increase in the odds of polypharmacy (OR 1.08; 95% CI 1.07, 1.09). Compared with responders with a BMI < 25, those who were overweight (BMI 25–30) had 1.50 times the odds of polypharmacy (OR 1.50; 95% CI 1.24, 1.81), and those with obesity (BMI ≥ 30) had 1.92 times the odds of polypharmacy (OR 1.92; 95% CI 1.60, 2.32). Responders in pre-9/11 protective service occupations had a 1.30 times the odds of polypharmacy compared with those in other occupations. There were also significant associations between several chronic WTC-certified conditions and polypharmacy. Responders with cancer (OR 1.30; 95% CI 1.12, 1.50), GERD (OR 1.71; 95% CI 1.47, 1.99), OAD (OR 2.24; 95% CI 1.91, 2.62), and URD (OR 1.85; 95% CI 1.60, 2.14) had higher odds of polypharmacy compared with responders without these conditions. In contrast, male responders (OR 0.81; 95% CI 0.68–0.97) and those in pre-9/11 construction occupations (OR 0.73; 95% CI 0.60–0.88) had lower odds of polypharmacy compared with female responders and those in other occupations, respectively. Anxiety, depression, and PTSD were not significantly associated with polypharmacy in the multivariable model.

**Table 2:** Bivariate associations of cohort characteristics with polypharmacy.

Characteristics	Medication count < 5; N = 3125 (%)	Medication count ≥ 5; N = 3841 (%)	p-value
Age, median (IQR)	57 (53, 61)	59 (55, 65)	<0.001
Female sex (%)	357 (11.4)	588 (15.3)	<0.001
Race/ethnicity (%)			
White	1978 (63.3)	2373 (61.8)	0.3
Black	422 (13.5)	519 (13.5)	
Hispanic	634 (20.3)	846 (22.0)	
Other	91 (2.9)	103 (2.7)	
Enrollment year (%)			
2002-2005	1148 (36.7)	1536 (40.0)	0.002
2006-2008	732 (23.4)	926 (24.1)	
2009-present	1245 (39.8)	1379 (35.9)	
Exposure Severity (%)			
Low	451 (14.4)	540 (14.1)	0.8
Intermediate	1971 (63.1)	2413 (62.8)	
High or Very High	703 (22.5)	888 (23.1)	
Pre-9/11 occupation (%)			
Construction	585 (18.7)	794 (20.7)	<0.001
Protective	1788 (57.2)	1876 (48.8)	
Other	483 (15.5)	785 (20.4)	
Maintenance and Repair	269 (8.6)	386 (10.0)	
Current smoker (%)	199 (6.4)	224 (5.8)	<0.001
Former smoker (%)	849 (27.2)	1402 (36.5)	
Never Smoker (%)	2077 (66.5)	2215 (57.7)	
Alcohol use (%)			
None	664 (21.2)	1258 (32.8)	<0.001
<1 drink per week	1865 (59.7)	2019 (52.6)	
≥1 drink per week	596 (19.1)	564 (14.7)	
BMI (%)			
<25	450 (14.4)	394 (10.3)	<0.001
25-30	1340 (42.9)	1391 (36.2)	
>30	1335 (42.7)	2056 (53.5)	
Chronic conditions (%)			
Hypertension	1239 (39.6)	2689 (70.0)	<0.001
Diabetes Mellitus	318 (10.2)	1177 (30.6)	<0.001
Obstructive Airway Disease	354 (11.3)	1555 (40.5)	<0.001
Sleep Apnea	711 (22.8)	2011 (52.4)	<0.001
Upper Respiratory Disease	869 (27.8)	2363 (61.5)	<0.001
Cancer	498 (15.9)	865 (22.5)	<0.001
GERD	631 (20.2)	2031 (52.9)	<0.001
PTSD	101 (3.2)	482 (12.5)	<0.001
Depression	49 (1.6)	283 (7.4)	<0.001
Anxiety Disorder	60 (1.9)	213 (5.5)	<0.001
Self-reported health (%)			
Excellent/very good/good	2597 (83.1)	2096 (54.6)	<0.001
Fair/poor	528 (16.9)	1745 (45.4)	
*Health limits accomplishments (%)	860 (27.5)	2129 (55.4)	<0.001
**Health limits moderate activities (%)	205 (6.6)	882 (23.0)	<0.001
Pain limits work (%)			
Not at all	1330 (42.6)	780 (20.3)	<0.001
A little bit	857 (27.4)	922 (24.0)	
Moderately	549 (17.6)	987 (25.7)	
Quite a bit/Extremely	389 (12.4)	1152 (30.0)	
Short term memory problems (%)			
Not at all	928 (29.7)	716 (18.6)	<0.001
A little	1304 (41.7)	1384 (36.0)	
Moderately	500 (16.0)	852 (22.2)	
Quite a bit/extremely	393 (12.6)	889 (23.1)	
WTC FI-Clinical (deficit count)			
Median (IQR)	2 (1, 5)	6 (3, 10)	<0.001

*“During the past 4 weeks, have you had any of the following problems with your work or regular daily activities as a result of your physical health: Accomplished less than you would like?”.

**“Does your health now limit you in: Moderate activities, such as moving a table, pushing a vacuum cleaner, bowling, or playing golf”.

**Fig 3:**
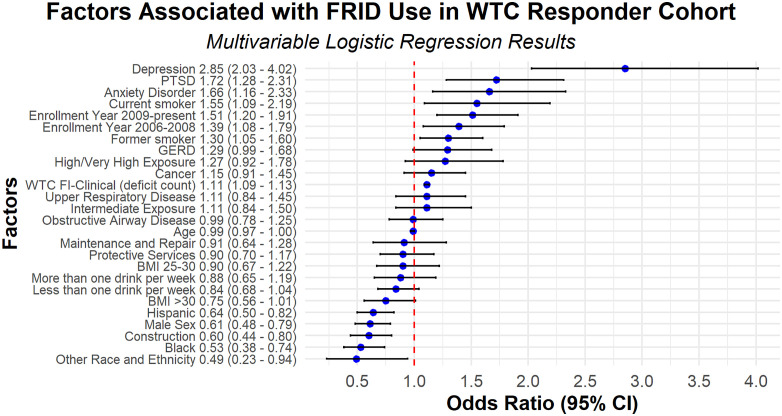
This forest plot presents the multivariable logistic regression analysis of factors associated with FRID use in the WTC Responder Cohort. Odds ratios and their corresponding 95% confidence intervals are shown for each factor.

Among the cohort, 7.6% of participants used FRIDs (Table 3). Table 3 displays bivariate associations between various cohort characteristics and FRID use. In the bivariate analysis, frailty was associated with FRID use. FRID use was also associated with older age, female sex, former or current smoking, occupations in maintenance and repair and other roles, and chronic conditions including hypertension, OAD, sleep apnea, URD, GERD, PTSD, depression, and anxiety. Fig 3 presents the results of the multivariable model that evaluates factors associated with FRID use. In the multivariable model, frailty was independently associated with FRID use. Each additional deficit in the WTC FI-Clinical was associated with an 11% increase in the odds of FRID use (OR 1.11; 95% CI 1.09, 1.13). There were also significant associations between several cohort characteristics and FRID use. Female responders had 1.64 times the odds of FRID use compared with male responders (OR 1.64; 95% CI 1.27, 2.08). Compared to never smokers, the odds of FRID use were 1.55 times higher among current smokers (OR 1.55; 95% CI 1.09, 2.19) and 1.30 times higher among former smokers (OR 1.30; 95% CI 1.05, 1.60). There were also significant associations between several WTC-certified conditions and FRID use. Responders with anxiety disorder (OR 1.66; 95% CI 1.16, 2.33), depression (OR 2.85; 95% CI 2.03, 4.02), and PTSD (OR 1.72; 95% CI 1.28, 2.31) had higher odds of FRID use compared with responders without these conditions. In contrast, race/ethnicity was inversely associated with FRID use. Compared to White participants, the odds of FRID use were.53 times lower among Black participants (OR 0.53; 95% CI 0.38, 0.74) and.64 times lower among Hispanic participants (OR 0.64; 95% CI 0.50, 0.82).

**Table 3:** Bivariate associations of cohort characteristics with FRID use.

Characteristics	No FRID use N = 6437 (%)	FRID use	p-value
		N = 529 (%)	
Age, median (IQR)	58 (54, 63)	59 (55, 64)	0.023
Female sex (%)	831 (12.9)	114 (21.6)	<0.001
Race/ethnicity (%)			
White	3995 (62.1)	356 (67.3)	0.007
Black	893 (13.9)	48 (9.1)	
Hispanic	1365 (21.2)	115 (21.7)	
Other	184 (2.9)	10 (1.9)	
Enrollment year (%)			
2002-2005	2497 (38.8)	187 (35.3)	0.3
2006-2008	1521 (23.6)	137 (25.9)	
2009-present	2419 (37.6)	205 (38.8)	
Exposure Severity (%)			
Low	926 (14.4)	65 (12.3)	0.2
Intermediate	4056 (63.0)	328 (62.0)	
High or Very High	1455 (22.6)	136 (25.7)	
Pre-9/11 occupation (%)			
Construction	1279 (19.9)	100 (18.9)	<0.001
Protective Services	3432 (53.3)	232 (43.9)	
Other	1136 (17.6)	132 (25.0)	
Maintenance and Repair	590 (9.2)	65 (12.3)	
Current smoker (%)	372 (5.8)	51 (9.6)	<0.001
Former smoker (%)	2049 (31.8)	202 (38.2)	
Never smoker (%)	4016 (62.4)	276 (52.2)	
Alcohol use (%)			
None	1717 (26.7)	205 (38.8)	<0.001
<1 drink per week	3634 (56.5)	250 (47.3)	
≥1 drink per week	1086 (16.9)	74 (14.0)	
BMI (%)			
<25	766 (11.9)	78 (14.7)	0.15
25-30	2533 (39.4)	198 (37.4)	
>30	3138 (48.7)	253 (47.8)	
Chronic conditions (%)			
Hypertension	3602 (56.0)	326 (61.6)	0.01
Diabetes mellitus	1371 (21.3)	124 (23.4)	0.2
Obstructive Airway Disease	1678 (26.1)	231 (43.7)	<0.001
Sleep Apnea	2432 (37.8)	290 (54.8)	<0.001
Upper Respiratory Disease	2889 (44.9)	343 (64.8)	<0.001
Cancer	1242 (19.3)	121 (22.9)	0.05
GERD	2347 (36.5)	315 (59.5)	<0.001
PTSD	435 (6.8)	148 (28.0)	<0.001
Depression	219 (3.4)	113 (21.4)	<0.001
Anxiety Disorder	211 (3.3)	62 (11.7)	<0.001
Self-report general health (%)			
Excellent/very good/good	4467 (69.4)	226 (42.7)	<0.001
Fair/poor	1970 (30.6)	303 (57.3)	
*Health limits accomplishments (%)	2629 (40.8)	360 (68.1)	<0.001
**Health limits moderate activities (%)	911 (14.2)	176 (33.3)	<0.001
Pain limits work (%)			
Not at all	2039 (31.7)	71 (13.4)	<0.001
A little	1695 (26.3)	84 (15.9)	
Moderately	1394 (21.7)	142 (26.8)	
Quite a bit/Extremely	1309 (20.3)	232 (43.9)	
Short term memory problems (%)			
Not at all	1585 (24.6)	59 (11.2)	<0.001
A little	2541 (39.5)	147 (27.8)	
Moderately	1230 (19.1)	122 (23.1)	
Quite a bit/extremely	1081 (16.8)	201 (38.0)	
WTC FI-Clinical (deficit count)			
Median (IQR)	3 (1, 8)	9 (4, 13)	<0.001

*“During the past 4 weeks, have you had any of the following problems with your work or regular daily activities as a result of your physical health: Accomplished less than you would like?”.

**“Does your health now limit you in: Moderate activities, such as moving a table, pushing a vacuum cleaner, bowling, or playing golf”.

Given the reduction from 11,605 eligible responders to 6,966 complete cases, we assessed potential bias by comparing baseline characteristics of included vs excluded responders (S1 Table). Multiple characteristics differed statistically, but absolute differences were small and rates of polypharmacy and FRID use were similar, suggesting the analytic cohort is broadly comparable. Also, we performed multiple-imputation sensitivity analyses. Results from the multiple imputation models (n = 11,605) closely matched the complete-case estimates: odds ratios and 95% CIs were very similar, directions were unchanged, and key associations remained stable in magnitude. A few coefficients crossed the p = 0.05 threshold under multiple imputation (consistent with greater precision from the larger sample), but effect sizes were essentially unchanged (S1 and S2 Figs).

## Discussion

The WTC disaster occurred over two decades ago, and responders are entering middle and older age. Recognizing the established risks associated with polypharmacy in aging populations, our study examines polypharmacy patterns in this aging cohort to identify potential health risks and areas for intervention. Our findings underscore the urgency of addressing this issue in the WTC responder population, as we observed a strikingly high prevalence of polypharmacy compared to the general population: 55% of participants met the criteria for polypharmacy (≥ 5 medications) and 21% for hyper-polypharmacy (≥ 10 medications). These findings point to significant medication burden at a relatively younger age within this unique cohort.

Compared to reported polypharmacy rates in the general US population, WTC responders demonstrate a similar prevalence at a younger age [11–14,32]. Recent studies in the general population found that polypharmacy rates increase with age, with approximately 36.8–44.1% of adults aged 65 and older, and up to 67.1% of adults aged 62–85 years reporting polypharmacy [11–14,32]. In contrast, WTC responders, with a median age of approximately 58 years, exhibit similar or higher prevalence rates, suggesting that WTC exposure and its sequelae may accelerate the development of chronic conditions requiring pharmacotherapy. This trend aligns with prior research indicating potential accelerated aging in WTC responders, as evidenced by findings of cognitive impairment [33–35] and increased frailty [7,8,36]. These factors likely contribute to the elevated polypharmacy rates observed, further highlighting the long-term health impacts of WTC exposure.

We also found that polypharmacy was independently associated with frailty. Specifically, each additional deficit on the frailty index increased the likelihood of polypharmacy by approximately 15%, highlighting the relationship between medication burden and physical vulnerability in this unique population. This is consistent with prior studies examining the relationship between frailty and polypharmacy, although the relationship could be bidirectional [19]. Given the strong association between these factors, optimizing medication use and reducing polypharmacy could be a potential target for mitigating frailty. Evidence from a systematic review of deprescribing interventions in older adults with frailty suggests that targeted deprescribing may reduce frailty scores and improve other clinical outcomes, though studies are limited in number and scope [37]. Whether such interventions are applicable to WTC responders with their unique environmental exposure history warrants further investigation.

Additionally, identifying high-risk groups within the cohort could help in tailoring interventions to those who may benefit most. For example, women in our cohort appear to be at increased risk for polypharmacy, FRID use and frailty [7], a trend consistent with broader literature that reports higher rates of polypharmacy [38], frailty [39], FRID use [40] among women compared to men. A review of nine studies identified gender as a risk factor for polypharmacy but noted that this effect tends to level out in older age groups [38]. This aligns with our findings, as our cohort is relatively younger. However, polypharmacy also remains highly prevalent among male WTC responders, underscoring the need for interventions targeting both sexes. Another high-risk group in our cohort includes responders with obesity. Prior studies have associated obesity with polypharmacy, as obesity often contributes to the development of chronic diseases, which in turn increases the need for multiple medications to manage associated comorbidities [41,42]. Additionally, certain pre-9/11 occupations were associated with polypharmacy. Construction was associated with polypharmacy in the bivariate analysis, but this shifted to protective services in the multivariable model. Future research should investigate how current occupational roles, occupational exposures, and lifestyle factors may have contributed to the development of medical conditions and the subsequent prescribing of medications. Importantly, some medications may hinder job performance and safety, emphasizing the need for careful medication management to protect both worker health and public safety. Given the elevated vulnerability to polypharmacy among women, responders with obesity, and those in occupations like protective services, tailored interventions for these high-risk groups could play a critical role in mitigating adverse health outcomes associated with polypharmacy and supporting occupational safety and overall well-being in this cohort.

We also found that specific WTC-related conditions are associated with polypharmacy and use of FRIDs. The unique environmental and psychological exposures experienced by WTC responders likely contributed to the development of multiple chronic health conditions, which in turn necessitated complex medication regimens. In this study, we found that conditions such as respiratory diseases, cancer, and GERD were associated with polypharmacy independent of frailty, suggesting that WTC-related conditions play a key role in driving medication use in this study sample. Psychiatric conditions common among WTC responders, such as anxiety, depression, and PTSD, further reflect the long-term health impacts of WTC exposures. While these conditions were not associated with polypharmacy in our study, they were associated with an increased risk of FRID use. Many of these psychiatric WTC-related conditions are treated with FRIDs such as sedative-hypnotics, tricyclic antidepressants, and antipsychotics, necessitating careful consideration of their side-effect profiles as this cohort ages [43]. It is important to recognize that polypharmacy is not inherently inappropriate but often reflects the complexity of medical needs in populations like WTC responders. Providers must use clinical judgement as well as partner with patients to discuss benefits and risks of specific medications, especially those considered to be FRIDs [16]. Tailored medication management strategies are essential to minimize harmful side effects while ensuring effective treatment of their chronic and psychiatric conditions. Adopting a balanced approach that considers potential risks and benefits, along with patient preferences and goals, may be valuable when guided by established clinical tools, such as the Beers Criteria, for prescribing in older adults [16,44].

This study has several limitations. First, we included a subset of WTC responder cohort with available data on medication use and frailty rather than the entire WTC responder population. However, our included sample is comparable to the excluded subset across key characteristics (S1 Table). Also, findings from the sensitivity analyses suggest our findings are robust when including the rest of the sample through multiple imputation (S1 and S2 Figs). Second, because this analysis did not include a control group, our study design does not allow us to isolate WTC responder–specific effects from the observed associations. Third, medication use was ascertained via self-report through structured interviews conducted by clinical staff. This is similar to prior studies examining medication use in large populations [12–14]. The structured interviews in our study included OTC medications, which can interact with prescriptions and increase fall risk. This is particularly important as older adults often use both types of therapies, making it essential to address these interactions and their associated risks [32,45]. Finally, it is important to note that the WTC responder cohort represents a group with specific environmental exposures and health risks related to 9/11. This may limit generalizability to the general population, though our findings may translate to other populations that have environmental exposures with health impacts of similar nature to the WTC disaster [46–48].

Our findings suggest that WTC responders, with their significant environmental and psychological exposure, are at high risk for polypharmacy and use of potentially harmful medications. Our work underscores the current need for approaches to optimize medication use in this population, which includes input from key stakeholders such as responders and healthcare professionals. As the WTC responder population ages, the goals of the CDC WTC Health Program remain critical in supporting their long-term health and well-being. Future research efforts are needed to better understand the risk of polypharmacy on the development and progression of frailty, as well as on poor health outcomes, to guide effective interventions as the cohort ages.

## Supporting information

S1 Fig Multiple imputation sensitivity analysis for polypharmacy. This forest plot compares odds ratios and 95% confidence intervals from the multiple-imputation and complete-case multivariable logistic regression models for factors associated with polypharmacy in the WTC Responder Cohort.
(TIF)

S2 Fig Multiple imputation sensitivity analysis for FRID use. This forest plot compares odds ratios and 95% confidence intervals from the multiple-imputation and complete-case multivariable logistic regression models for factors associated with FRID use in the WTC Responder Cohort.
(TIF)

S1 Table Comparison of Characteristics Between Included and Excluded Study Participants Due to Missing Values on Key Variables. This table compares characteristics of participants included in the study (N = 6,966) and those excluded due to missing data (N = 4,639).
(DOCX)

S2 Table Factors associated with polypharmacy by multivariable logistic regression. This table presents the multivariable logistic regression analysis of factors associated with polypharmacy (≥5 concurrent medications). Odds ratios, 95% confidence intervals, and p-values are reported.
(DOCX)

S3 Table Factors associated with FRID use by multivariable logistic regression. This table presents the multivariable logistic regression analysis of factors associated with the use of FRIDs. Odds ratios, 95% confidence intervals, and p-values are reported.
(DOCX)
