# Supplementary figures and images for: Polypharmacy and frailty among aging World Trade Center responders
Source: PLoS One. 2025 Dec 4;20(12):e0337391. doi: 10.1371/journal.pone.0337391 (PMC12677467; doi:10.1371/journal.pone.0337391)

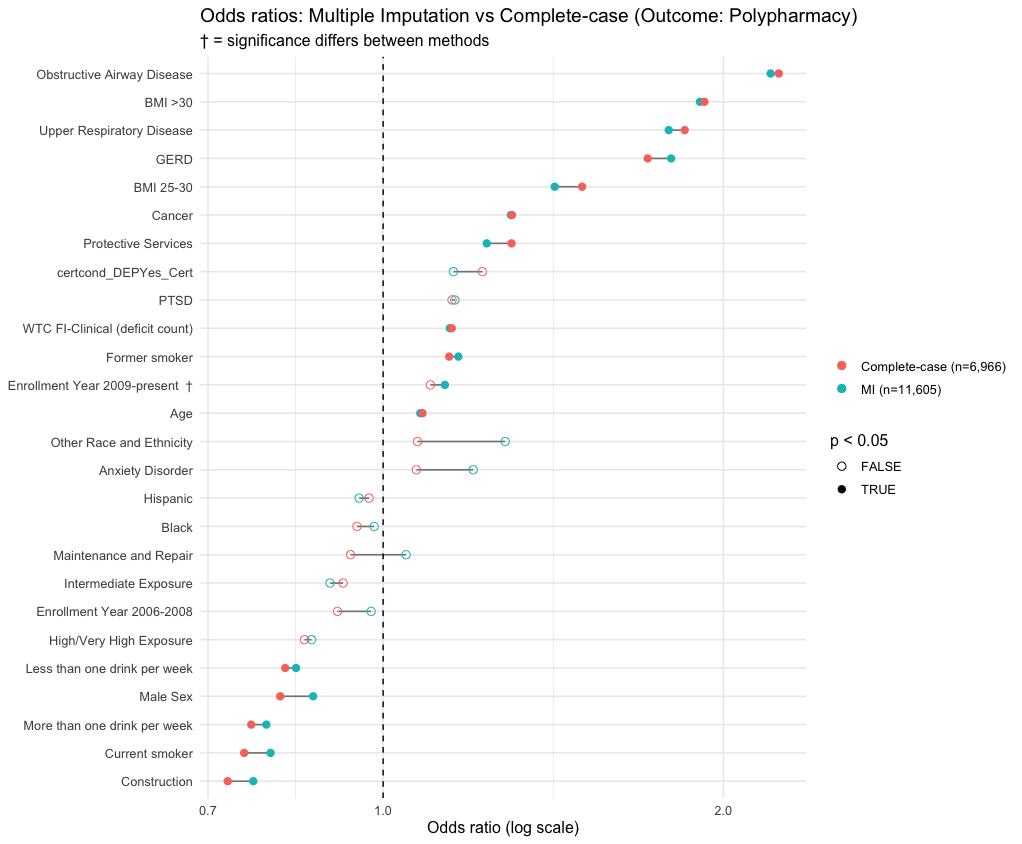

Supplement: S1 Fig — This forest plot compares odds ratios and 95% confidence intervals from the multiple-imputation and complete-case multivariable logistic regression models for factors associated with polypharmacy in the WTC Responder Cohort. (TIF) [file pone.0337391.s001.tif]

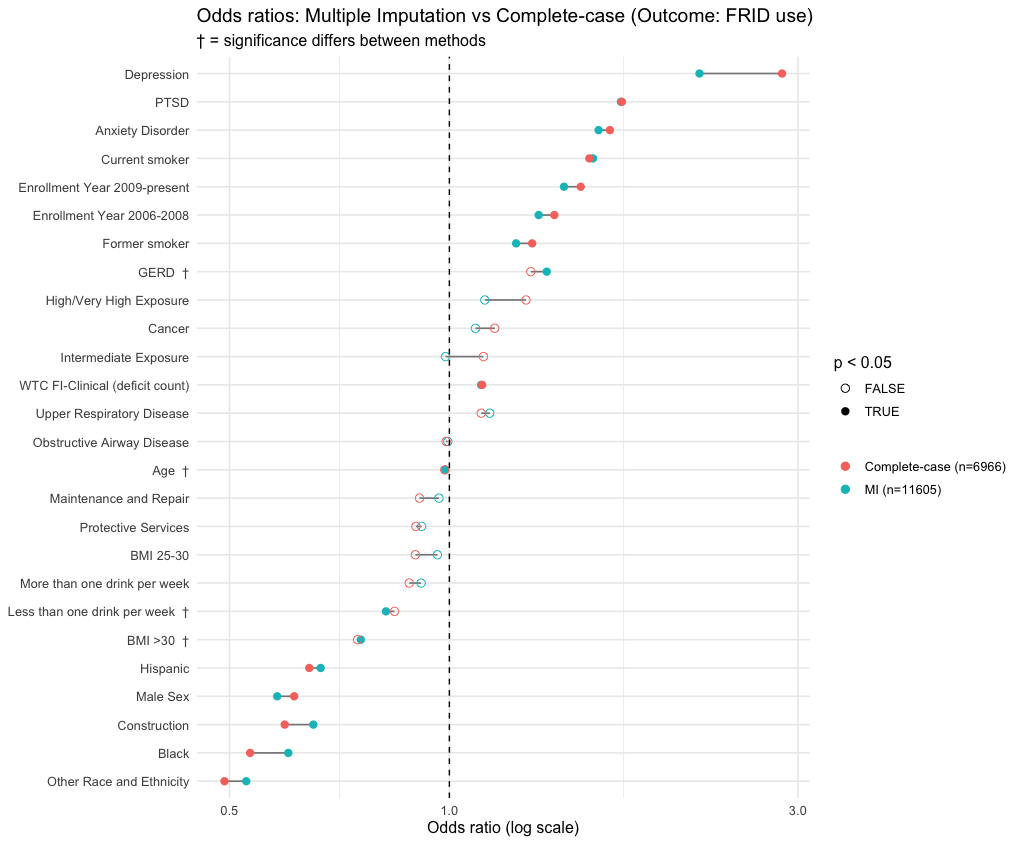

Supplement: S2 Fig — This forest plot compares odds ratios and 95% confidence intervals from the multiple-imputation and complete-case multivariable logistic regression models for factors associated with FRID use in the WTC Responder Cohort. (TIF) [file pone.0337391.s002.tif]
