# Supplementary material for: Polypharmacy and frailty among aging World Trade Center responders
Source: PLoS One. 2025 Dec 4;20(12):e0337391. doi: 10.1371/journal.pone.0337391 (PMC12677467; doi:10.1371/journal.pone.0337391)
Supplement: S1 Table — This table compares characteristics of participants included in the study (N = 6,966) and those excluded due to missing data (N = 4,639). (DOCX) [file pone.0337391.s003.docx]

Table S1: Comparison of Characteristics Between Included and Excluded Study Participants Due to Missing Values on Key Variables

| Complete Variables | | | |
| --- | --- | --- | --- |
| Variable | No, N = 4,639 | Yes, N = 6,966 | P-value |
| Polypharmacy Level |  |  |  |
| <5 Medications | 2,072 (45%) | 3,125 (45%) | 0.8 |
| ≥5 Medications | 2,567 (55%) | 3,841 (55%) |  |
| Fall-Risk Increasing Drugs Count |  |  |  |
| 0 | 4,268 (92%) | 6,437 (92%) | 0.3 |
| 1 | 322 (6.9%) | 451 (6.5%) |  |
| 2 | 40 (0.9%) | 71 (1.0%) |  |
| 3 | 9 (0.2%) | 7 (0.1%) |  |
| Age at Visit (years) |  |  |  |
| Median (IQR) | 59 (54, 65) | 58 (54, 63) | <0.001 |
| Sex |  |  |  |
| Female | 838 (18%) | 945 (14%) | <0.001 |
| Male | 3,801 (82%) | 6,021 (86%) |  |
| Race |  |  |  |
| Black | 630 (14%) | 941 (14%) |  |
| Hispanic | 1,066 (23%) | 1,480 (21%) | 0.003 |
| Other | 161 (3.5%) | 194 (2.8%) |  |
| White | 2,726 (59%) | 4,351 (62%) |  |
|  |  |  |  |
| Exposure severity |  |  |  |
| High/Very High | 905 (21%) | 1,591 (23%) | 0.011 |
| Intermediate | 2,786 (64%) | 4,384 (63%) |  |
| Low | 680 (16%) | 991 (14%) |  |
|  |  |  |  |
| Pre-9/11 Occupation |  |  |  |
| Construction | 996 (25%) | 1,379 (20%) | <0.001 |
| Protective | 1,720 (43%) | 3,664 (53%) |  |
| Other | 899 (22%) | 1,268 (18%) |  |
| Maintenance and Repair | 431 (11%) | 655 (9.4%) |  |
|  |  |  |  |
| Smoking Status |  |  |  |
| Current smoker | 310 (6.8%) | 423 (6.1%) |  |
| Former smoker | 1,517 (33%) | 2,251 (32%) | 0.10 |
| Never smoker | 2,722 (60%) | 4,292 (62%) |  |
|  |  |  |  |
| Alcohol Use |  |  |  |
| Less than one drink per week | 2,330 (51%) | 3,884 (56%) | <0.001 |
| More than one drink per week | 632 (14%) | 1,160 (17%) |  |
| None/non-drinker | 1,581 (35%) | 1,922 (28%) |  |
|  |  |  |  |
