# Supplementary material for: Polypharmacy and frailty among aging World Trade Center responders
Source: PLoS One. 2025 Dec 4;20(12):e0337391. doi: 10.1371/journal.pone.0337391 (PMC12677467; doi:10.1371/journal.pone.0337391)
Supplement: S2 Table — This table presents the multivariable logistic regression analysis of factors associated with polypharmacy (≥5 concurrent medications). Odds ratios, 95% confidence intervals, and p-values are reported. (DOCX) [file pone.0337391.s004.docx]

Table S2. Factors associated with polypharmacy by multivariable logistic regression

| Characteristic | OR^1^ | 95% CI^1^ | p-value |
| --- | --- | --- | --- |
| Age | 1.08 | 1.07, 1.09 | <0.001 |
| Male Sex (ref: female) | 0.81 | 0.68, 0.97 | 0.018 |
| Race (ref: white) |  |  |  |
| *Black* | 0.95 | 0.80, 1.12 | 0.5 |
| *Hispanic* | 0.97 | 0.84, 1.13 | 0.7 |
| *Other* | 1.07 | 0.76, 1.50 | 0.7 |
| Enrollment year (ref: 2002-2005) |  |  |  |
| *2006-2008* | 0.91 | 0.79, 1.06 | 0.2 |
| *2009-present* | 1.10 | 0.96, 1.26 | 0.2 |
| Exposure severity (Ref: low) |  |  |  |
| *High/Very High* | 0.85 | 0.71, 1.03 | 0.10 |
| *Intermediate* | 0.92 | 0.78, 1.09 | 0.3 |
| Pre-9/11 occupation (Ref: other) |  |  |  |
| *Construction* | 0.73 | 0.60, 0.88 | 0.001 |
| *Protective* | 1.30 | 1.10, 1.53 | 0.002 |
| Maintenance and Repair | 0.94 | 0.74, 1.18 | 0.6 |
| Smoking status (Ref: never) |  |  |  |
| *Current smoker* | 0.75 | 0.59, 0.96 | 0.022 |
| *Former smoker* | 1.14 | 1.01, 1.30 | 0.035 |
| Alcohol use (Ref: none) |  |  |  |
| *Less than one drink per week* | 0.82 | 0.72, 0.94 | 0.004 |
| *More than one drink per week* | 0.76 | 0.64, 0.91 | 0.003 |
| BMI (ref: <25) |  |  |  |
| *25-30* | 1.50 | 1.24, 1.81 | <0.001 |
| *>30* | 1.92 | 1.60, 2.32 | <0.001 |
| Anxiety Disorder | 1.07 | 0.76, 1.52 | 0.7 |
| Cancer | 1.30 | 1.12, 1.50 | <0.001 |
| Depression | 1.22 | 0.82, 1.85 | 0.3 |
| GERD | 1.71 | 1.47, 1.99 | <0.001 |
| Obstructive Airway Disease | 2.24 | 1.91, 2.62 | <0.001 |
| PTSD | 1.15 | 0.86, 1.54 | 0.3 |
| Upper Respiratory Disease | 1.85 | 1.60, 2.14 | <0.001 |
| WTC FI-Clinical (deficit count) | 1.15 | 1.13, 1.17 | <0.001 |
| ^1^OR = Odds Ratio, CI = Confidence Interval | | | |
