# Supplementary material for: Polypharmacy and frailty among aging World Trade Center responders
Source: PLoS One. 2025 Dec 4;20(12):e0337391. doi: 10.1371/journal.pone.0337391 (PMC12677467; doi:10.1371/journal.pone.0337391)
Supplement: S3 Table — This table presents the multivariable logistic regression analysis of factors associated with the use of FRIDs. Odds ratios, 95% confidence intervals, and p-values are reported. (DOCX) [file pone.0337391.s005.docx]

Table S3. Factors Associated with FRID use by multivariable logistic regression

| Characteristic | OR^1^ | 95% CI^1^ | p-value |
| --- | --- | --- | --- |
| Age | 0.99 | 0.97, 1.00 | 0.065 |
| Male Sex (ref: female) | 0.61 | 0.48, 0.79 | <0.001 |
| Race (ref: white) |  |  |  |
| *Black* | 0.53 | 0.38, 0.74 | <0.001 |
| *Hispanic* | 0.64 | 0.50, 0.82 | <0.001 |
| *Other* | 0.49 | 0.23, 0.94 | 0.046 |
| Enrollment year (ref: 2002-2005) |  |  |  |
| *2006-2008* | 1.39 | 1.08, 1.79 | 0.009 |
| *2009-present* | 1.51 | 1.20, 1.91 | <0.001 |
| Exposure severity (ref: Low) |  |  |  |
| *High/Very High* | 1.27 | 0.92, 1.78 | 0.15 |
| *Intermediate* | 1.11 | 0.84, 1.50 | 0.5 |
| Pre-9/11 occupation (ref: other) |  |  |  |
| *Construction* | 0.60 | 0.44, 0.80 | <0.001 |
| *Protective* | 0.90 | 0.70, 1.17 | 0.4 |
| Maintenance and Repair | 0.91 | 0.64, 1.28 | 0.6 |
| Smoking status (ref: none) |  |  |  |
| *Current smoker* | 1.55 | 1.09, 2.19 | 0.013 |
| *Former smoker* | 1.30 | 1.05, 1.60 | 0.014 |
| Alcohol use (ref: none) |  |  |  |
| *Less than one drink per week* | 0.84 | 0.68, 1.04 | 0.11 |
| *More than one drink per week* | 0.88 | 0.65, 1.19 | 0.4 |
| BMI (ref: <25) |  |  |  |
| *25-30* | 0.90 | 0.67, 1.22 | 0.5 |
| *>30* | 0.75 | 0.56, 1.01 | 0.054 |
| Anxiety Disorder | 1.66 | 1.16, 2.33 | 0.004 |
| Cancer | 1.15 | 0.91, 1.45 | 0.2 |
| Depression | 2.85 | 2.03, 4.02 | <0.001 |
| GERD | 1.29 | 0.99, 1.68 | 0.056 |
| Obstructive Airway Disease | 0.99 | 0.78, 1.25 | >0.9 |
| PTSD | 1.72 | 1.28, 2.31 | <0.001 |
| Upper Respiratory Disease | 1.11 | 0.84, 1.45 | 0.5 |
| WTC FI-Clinical (deficit count) | 1.11 | 1.09, 1.13 | <0.001 |
| ^1^OR = Odds Ratio, CI = Confidence Interval | | | |
